# A structural equation model of falls at home in individuals with chronic stroke, based on the international classification of function, disability, and health

**DOI:** 10.1371/journal.pone.0231491

**Published:** 2020-04-10

**Authors:** Kalaya Kongwattanakul, Vimonwan Hiengkaew, Chutima Jalayondeja, Yothin Sawangdee

**Affiliations:** 1 Faculty of Physical Therapy, Mahidol University, Salaya, Nakhon Pathom, Thailand; 2 Institute for Population and Social Research, Mahidol University, Salaya, Nakhon Pathom, Thailand; University of Birmingham, UNITED KINGDOM

## Abstract

**Purpose:**

To use structural equation model (SEM) to explain falls at home in individuals with chronic stroke, based on the International Classification of Functioning, Disability and Health (ICF).

**Materials and methods:**

A cross sectional observation study was conducted in home-dwelling individuals with chronic stroke (N = 236; 148 non-fallers, 88 fallers). Participants were assessed; structural impairments using Modified Ashworth Scale, Fugl-Meyer Assessment upper (FMA-UE), lower (FMA-LE), and sensory function, ankle plantarflexor strength; activity limitations using Timed Up and Go Test, Step Test, Berg Balance Scale, Barthel Index (BI); participation restrictions using Stroke Impact Scale-participation (SIS-P); and contextual factors using home hazard environments, home safety surroundings, risk behaviors, and Fall-related Self Efficacy. The measurement model was analyzed by confirmatory factor analysis. The SEM was conducted to analyze a structural model of falls at home.

**Results:**

FMA-UE was significantly (p<0.01) associated with FMA-LE, combining as one variable in the structural impairments. In the measurement model, variables were fit to their domains, except variables of contextual factors, but the ICF domains did not correspond to disability. A structural model of falls at home demonstrated a significant (p<0.01) direct path of contextual factors and activity limitations with falls at home. The structural impairments showed a significant (p<0.01) direct path with activity limitations. All variables, except BI, SIS-P and risk behaviors, related to their domains in the structural model.

**Conclusions:**

A structural model of falls at home proposes contextual factors being the strongest association with falls at home that home hazard environments seem the most influence in its domain. The activity limitations presented by balance ability are directed to falls at home. The structural impairments are associated with falls at home through activity limitations. Home assessment to decrease home hazard environments is suggested to prevent falls at home for individuals with chronic stroke.

## Introduction

Individuals with stroke have long-term disability and are at high risk of falls. A rate of falls was 8.7 [1] or 1.3–6.5 falls/person/year [2], otherwise, 3.57 [3], or about 1.77 times a year [4]. Falls are associated with several factors following stroke, for example, muscle strength [5], muscle tone [6, 7], postural balance [4, 5], activities of daily living [3, 8–10], going out into community for shopping [10], tripping hazards [11], confidence in mobility and balance [4], depression [3, 5], number of medication [5]. With intrinsic or extrinsic factors, individuals with stroke are susceptible to falls.

Cause of falls is an interaction of various risk factors. Individuals with stroke contain a number of fall risk factors that can reciprocally link, for instance, lower limb performance and mobility [12], motor function and balance [13], balance and walking and participation restrictions [14], self-efficacy and physical activity [15], or environment and frequency of walking [16]. The International Classification of Functioning, Disability and Health (ICF) is a framework integrating medical and social components to describe health condition of people [17] and can be a possible framework for establishing and guiding management for patients [18, 19]. A previous study demonstrated the use of the ICF for selecting assessment tools associating with falls in individuals with stroke, for example Fugl-Meyer Assessment lower extremity function (FMA-LE) for structural impairments, Berg Balance Scale (BBS) for activity limitations [20]. However, several measurement tools for the ICF structural impairments [21], activity limitations [22], and participation restrictions have been proposed [23]. Selection of tools for the ICF structural impairments is suggested to a specific determination [21]. Among outcome measures evaluating the ICF body functions, the Fugl-Meyer Assessment (FMA) and Modified Ashworth Scale (MAS) are recommended tools [21]. According to the ICF core sets for stroke, muscle tone and strength are components of body structures [24]. Spasticity relates to falls in individuals with stroke [6, 7]. The MAS examines muscle tone [25] and shows moderate to substantial agreement for inter-and intra-rater for elbow flexor and ankle plantaflexor [26]. In addition to tone of ankle plantarflexor, the strength of the muscle is important for walking [27–29], a category of component activities and participation [24], and associates with fear of falling in individuals with stroke [30]. There is no tool proposed to measure muscle strength in the ICF structural impairments [21]. Manual muscle test is a common method assessing muscle strength in clinic but it is inaccurate [31]. Handheld dynamometer is an approach providing objective measure of muscle force [32] and is usually used in research [33]. It shows high intra-reliability [34] and good to high inter-reliability [35]. Some items in the ICF categories of component activities for individuals with stroke may relate to fall risk such as undertaking single or multiple tasks, carrying out daily routine, changing or maintaining position, transferring, moving, doing housework, or walking [24]. Numerous outcome measures is suggested to evaluate the ICF activities [22]. The BBS, Timed Up and Go Test (TUG), and Barthel Index (BI) seem to be consensus measurements in the ICF activity [22]. Although the TUG is slightly recognized among the BBS and BI for selecting to assess activity [22], it is shown to detect risk of falls in individuals with stroke [36]. A systematic review shows asymmetrical weight bearing following stroke associating with postural sway and demonstrates weak association between the asymmetrical weight bearing and the BBS [37]. A measurement tool that is the step test (ST) assessing weight bearing during standing [38] and correlating with balance and lower limb coordination [39] may explain falls in the ICF activity. For outcome measures of the ICF participation restrictions there is a variety of measurements and lack of consensus for measure [23]. The Stroke Impact Scale is one of outcome measures [23] consisting of multiple domain assessment that summed scores of each domain can be generated [40]. Thus, it decreases inconvenience in respondents and increases possibility of researchers [23]. Although there are various components in the ICF domains related to falls, a particular reason or a related cause of falls occurring in individuals with stroke has not yet explained and some tools appearing associating with falls have not been established for using in the ICF. Moreover, there is no suggestion of successful management for primary or secondary fall prevention in individuals with stroke [41]. Therefore, cause of falls in individuals with stroke is feasible to be explained by using the ICF.

However, the use of the ICF for explaining cause of falls in individuals with stroke is just a theoretical model based on structural impairments, activity limitations, participations restrictions, and contextual factors [17]. To test the complex multifactorial theoretical model and establish a model for describing cause of falls in individuals with stroke, structural equational model (SEM)-a statistical method-can be used [42]. Concerning place of falls, individuals with stroke are absolute exposure for falls in their home since they might spend more time at home or be more carefulness when they are outside of their home [4]. It was reported with a rate of 70% of falls occurring at home [10]. Therefore, the objective of the present study was to use the SEM to explain a model of falls at home, based on the ICF, in individuals with chronic stroke. The present study may contribute to understand cause of falls at home and may apply for falls prevention at home in individuals with chronic stroke.

## Methods

### Procedure

Ethical approval has been obtained from the Ethical Committee of Mahidol University (MU-IRB 2014/042.1903/COA No. 2014/060.0805). Clinical trial has been registered by Thai Clinical Trials Registry (TCTR20170723001). The study was conducted in accordance with the Declaration of Helsinki ethical principles for human experimentation.

A researcher together with a physical therapist visited individuals with stroke, who enrolled on the study, at home. The researcher asked participants to sign written inform consent, interviewed about age, weight, height, marital status, stroke onset, type of stroke, side of body weakness, comorbidity, performance of activities of daily living using the BI [43], impact of stroke on life using Stroke Impact Scale-Participation (SIS-P) [40], fall concern using Fall-related Self Efficacy (FES) [44], gait devices, receiving physical therapy, amount of falls at home and risk behaviors in the last 6 months, and surveyed home hazard and safety environments.

While the researcher surveyed home, a physical therapist assessed muscle tone by MAS [25] on elbow flexor (MAS-EF), and ankle plantarflexor (MAS-AP), evaluated motor function and sensation by FMA upper extremity function (FMA-UE), FMA-LE, and sensation function (FMA-S) [45], and measured ankle plantarflexor strength (APS) by dynamometer [46]. In addition, participants performed TUG [47], ST [38], and BBS [48].

The interview took 15 minutes, survey required 30 minutes, and assessments needed 45 minutes. All were done on the same day.

### Participants

Subjects were individuals with stroke who were recruited from a university hospital in Pathumthani or communities in Bangkok, Nonthaburi, Pathumthani, and Samut Prakarn. In the hospital a researcher directly contacted individuals with stroke in neurological outpatient clinic, while in the community a researcher asked public health or village volunteers for seeing individuals with stroke at home. Subjects were included if they (1) first stroke, (2) stroke more than 6 months, (3) either left or right side weakness, (4) able to walk 6 meters independently with or without gait device, (5) 2–4 scores of modified Ranking Scale (mRS), (6) ≥ 24 points of Mini Mental State Examination, and (6) fell or did not fall at home in the past 6 months prior to participation. Subjects were excluded if they (1) unilateral neglect, (2) unable to communicate, (3) visual impairments that could not remedy such as visual field deficits or double vision, (4) other neurological disorders, and (5) fractures in upper and lower limbs within 6 months prior to participation.

After recruitment, subjects were informed about the study and invited to enroll in the study. A researcher visited participants at home within 7 days after calling.

### Observed variables

#### Measure of falls at home

Participants themselves reported number, event, and reason of falls at home in the last 6 months prior to participation. Falls were defined as an episode of unintentionally coming to the ground or lower surface that did not result from dizziness, fainting, sustaining a violent blow, loss of consciousness, or other overwhelming external factors [49]. Participants reporting falls at home one or multiple times were in the group of falls at home, those who did not report were in the group of without falls at home.

#### Measures of structural impairment

*Modified Ashworth Scale (MAS) [25].* The MAS measures severity of muscle tone ranged from 0–4; 0 = no increase in tone, 1 = slight increase in tone and minimal resistance at the end of range of motion, 1+ = slight increase in tone and minimal resistance throughout less than half of the range of motion, 2 = more marked increase in tone, 3 = considerable increase in tone, and 4 = rigidity. The MAS scores were computed to 0–5 scale to include the score 1+ [50]. Elbow flexor and ankle plantarflexor muscle were chosen to evaluate muscle tone, that the MAS exhibits a consistent measure for both muscles [51, 52]. For data analysis, a score “0” was given if both muscles showed MAS = 0. A score “1” was given if any one of two muscles showed MAS ≥ 1. A score “2” was given if both muscles showed MAS ≥ 1.

*Fugl-Meyer Assessment (FMA) [45].* The FMA evaluates stroke recovery and has 3 points on each item. The present study used motor and sensory function domain. The motor function ranged from 0 (no function) to a maximum of 100 points (normal motor performance), with 66 points for the upper limb and 34 points for the lower limb. The sensory function ranged from 0 to a maximum of 24 points.

*Ankle plantarflexor strength (APS) [46].* The APS was assessed by a handheld dynamometer (Commander PowerTrak II, JTech Medical; Salt Lake, Utah, USA) in the affected and unaffected limb that the unit is Newton (N). In the supine position, participants performed 3 trials of a maximum isometric contraction of ankle plantarflexor, 5 seconds/trail. The instruction was “push, hold, hold, hold, and relax”. Participants were allowed a 15-second rest between trials and a 1-minute rest between limbs. The data of APS in the affected limb was calculated to percentage of the APS in the unaffected side.

#### Measure of activity limitations

*Timed Up and Go Test (TUG) [47].* The TUG evaluates functional mobility of rising from a chair, walking straight 3 meters, turning and sitting down. Participants did the test with preferred comfortable speed in standing up, walking, turning, and sitting down to avoid fall risk. The time to complete the task was recorded in seconds using a stopwatch (Professional stopwatch, Model No. JS-519; Shenzhen Junsd Industry Co, Ltd.).

*Step Test (ST) [38].* The ST assesses weight bearing ability while standing. Participants were asked to use the unaffected foot to step on and off a 7.5-cm height wooden step as fast as possible for 15 seconds. The number of steps was counted.

*Berg Balance Scale (BBS) [48].* The BBS measures 14 balance items, each on a 5-point scale ranging from 0–4 that 0 indicates inability to complete the task and 4 indicates ability to completely perform the task. Scores can range from 0 to 56.

*Barthel Index (BI) [43].* The BI assesses ability to do 10 activities of daily living. The total score ranges from 0 to a maximum 100 points with 0 indicating complete dependence and 100 showing independence.

#### Measure of participation restrictions

*Stroke Impact Scale-participation/role function (SIS-P) [40].* The SIS-P is one of 8 domains of Stroke Impact Scale. It consists of 8 items and is rated on difficulty with a task during the past 4 weeks on 5-point scale; 1 = all of the time, 2 = most of the time, 3 = some of the time, 4 = a little of the time, and 5 = none of the time. The score was summed and transformed to a score out of 100 using the formula: [(total score-8) / (40–8)] * 100. A higher score indicates a better perceived participation.

#### Measures of contextual factors

The contextual factors were composed of home hazard environments, home safety surroundings, risk behaviors, and fall concern. A questionnaire (See supporting information) was developed using a standardized procedure [53] to examine the first 3 variables. Three physical therapy faculties validated the questionnaire contents. A pilot study was used to determine which items were unclear or misleading. The index of item-objective congruence was 0.980 for both home hazard and safety environments, and 1.000 for risk behaviors. Reliability of risk behavior scale was excellent (α = 0.955).

*Home hazard environments and home safety surroundings (See supporting information)*. A number of home hazard environments and home safety surroundings were assessed, for example, slippery floors, obstacles on the floor, unstable chairs, the presence of handrails, stable objects, appropriate pathway width, night lights.

*Risk behaviors (See supporting information)*. Participants were examined how often they did the following risk activities at home in the past 6 months; hurry to sit down or stand up, sitting down on or standing up from floor, reaching an object higher than eyes level, picking objects from floor, doing bimanual task in standing position, dressing in standing position, doing activity that are never done alone, brisk walking, walking without gait aid despite using regularly, walking while carrying objects in both hands, and going to toilet at night without turning the light on. The frequency of risk performance was noted; 0 = never, 1 = sometimes, 2 = often, and 3 = always.

*Fall-related Self Efficacy (FES) [44].* The FES assesses fall concern while performing activities [44]. It consists of 13 items, each on 3 points; 0 = very concerned, 5 = fairly concerned, and 10 = not at all concerned. Scores can range from 0 to 130.

#### Intra-and inter-reliability of observed variables

A same researcher measured BI, home hazard environments, home safety surroundings, risk behaviors, and FES. The intra-reliability was good; BI [ICC(3,1) = 0.980], home hazard environments [ICC(3,1) = 0.890], home safety surroundings [ICC(3,1) = 0.899], risk behaviors [ICC(3,1) = 0.980], and FES [ICC(3,1) = 0.910].

Four physical therapists with more than 2-year experience in stroke physical therapy served as assessor for MAS, FMA, APS, TUG, ST, and BBS. They were blinded to fall history information. The inter-rater reliability was good; MAS-EF (Kappa range 0.840–1.00), MAS-AP (Kappa range 0.520–1.00), FMA-UE [ICC(2,1) = 0.989], FMA-LE [ICC(2,1) = 0.815], FMA-S [ICC(2,1) = 0.867], APS [ICC(2,1) = 0.751], TUG [ICC(2,1) = 0.943], ST [ICC(2,1) = 1.00], and BBS [ICC(2,1) = 0.965].

### Latent variables

Latent variables composed of structural impairments, activity limitations, participation restrictions, contextual factors, and disability.

### Statistical analysis

The statistical analyses were conducted with SPSS version 22 (SPSS Inc., Chicago, IL) together with STATA version 12 (StataCorp. 2011. Stata Statistical Software: Release 12. College Station, TX: StataCorp LP). Statistical significance was set at p < 0.05.

Normal distribution of characteristics of participants and observed variables data was tested with Kolmogorov–Smirnov test. The data was non-normal distribution, except age.

Comparison of characteristics of participants and variables between participants with and without falls at home was conducted with unpaired t-test, Mann-Whitney U-test, and Chi-square test, as appropriately. Numerical variables with a normal distribution were expressed as mean and standard deviation (SD), those with non-normal distribution were expressed as median and inter-quartile range (IQR). Nominal variables were presented as count and percentage (%)

**Fig 1:**
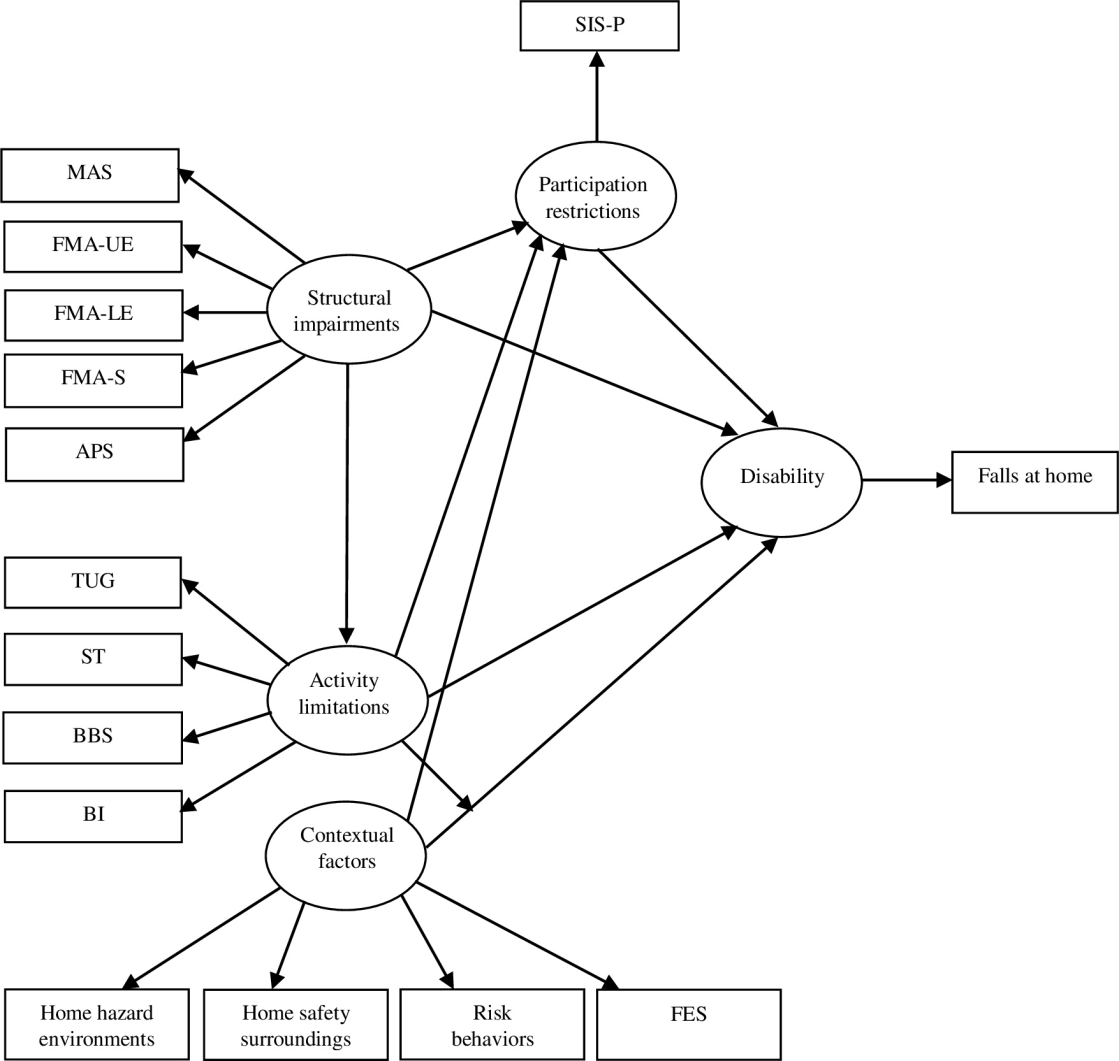
Hypothesis model for structural equational model analysis.

MAS: modified Ashworth scale; FMA-UE: Fugl-Meyer Assessment upper extremity function; FMA-LE: Fugl-Meyer Assessment lower extremity function; FMA-S: Fugl-Meyer Assessment sensory function; APS: Ankle plantarflexor strength; TUG: Timed Up and Go Test; ST: Step Test; BBS: Berg Balance Scale; BI: Barthel Index; SIS-P: Stroke Impact Scale-participation/role function; FES: Fall-related Self Efficacy.

The SEM which is covariance-based SEM technique was used for analyses of the hypothesized model based on the ICF background (Fig 1). The structural impairments were assessed by MAS, FMA-UE, FMA-LE, FMA-S, and APS. The activity limitations were determined by TUG, ST, BBS, and BI. The participation restrictions were evaluated by SIS-P. The contextual factors were measured by home hazard environments, home safety surroundings, risk behaviors, and FES. Individual or combination of structural impairments, activity limitations, participation restrictions, and contextual factors conducted disability in individuals with stroke, and finally, caused falls at home.

The first stage of the SEM analysis aimed to select observed variables into measurement model. The strength of association between any two observed variables among domains; MAS, FMA-UE, FMA-LE, FMA-S, APS, TUG, ST, BBS, BI, SIS-P, home hazard environments, home safety surroundings, risk behaviors, and FES was examined using Spearman’s rank test and multicollinearity. A correlation coefficient greater than 0.7 (r_s_>0.7), a tolerance less than 0.1 (t<0.1), or a variance inflation factor greater than 10 (VIF>10) indicates a strong relationship between variables that could unite to one single variable [54].

The second stage directed to analyze the fit of measurement model. The first order measurement model was to determine the fit of the selected variables for the domain of structural impairments, activity limitations, and contextual factors by using the confirmatory factor analysis (CFA). The SIS-P was directed to participation restrictions domain since it was the only one observed variable of the domain. The second order measurement model was to examine the fit of structural impairments, activity limitations, participation restrictions, and contextual factors for disability, also using the CFA. The CFA estimates factor loading and amount of measurement error.

The third stage aimed to examine the fit of structural model. The causal relationship of the selected and latent variables to the model of falls at home were determined by SEM. In the path diagram a single-headed arrow demonstrates the causal order between two variables with the arrow head pointing to the effect and its tail from the cause. Asymptotic distribution free was used to estimate factor loading, path coefficient, and amount of measurement error.

The fit of measurement and structural model was assessed with the p-value of Chi-square statistic greater than 0.05 (p>0.05), Chi-square/degree of freedom less than 2 (χ2/df<2), comparative fit index greater than 0.90 (CFI>0.90), root mean square error of approximation less than 0.05 (RMSEA<0.05), and standardized root mean residual less than 0.1(SRMR<0.1) [55]. A poor fit of the model was indicated by less than 3 indexes showing disagreement value. Then, the entire model was revised by the modification index by adding a covariance between error terms of variables to the model until the model fit indices were achieved [54, 55].

To control type I error inflation in the SEM, the false discovery rate controlling step-up Bonferroni was used to determine whether the alpha level was less than 0.05 (p<0.05).

The minimal sample size for the study was at least 154 cases based on power of analysis, 100 based on model complexity, 200 based on estimation technique, and 330 based on number of observed variables. The sample of 236 individuals with stroke in the study was plenty to do SEM, according to the recommendation [54].

## Results

### Participants

**Fig 2:**
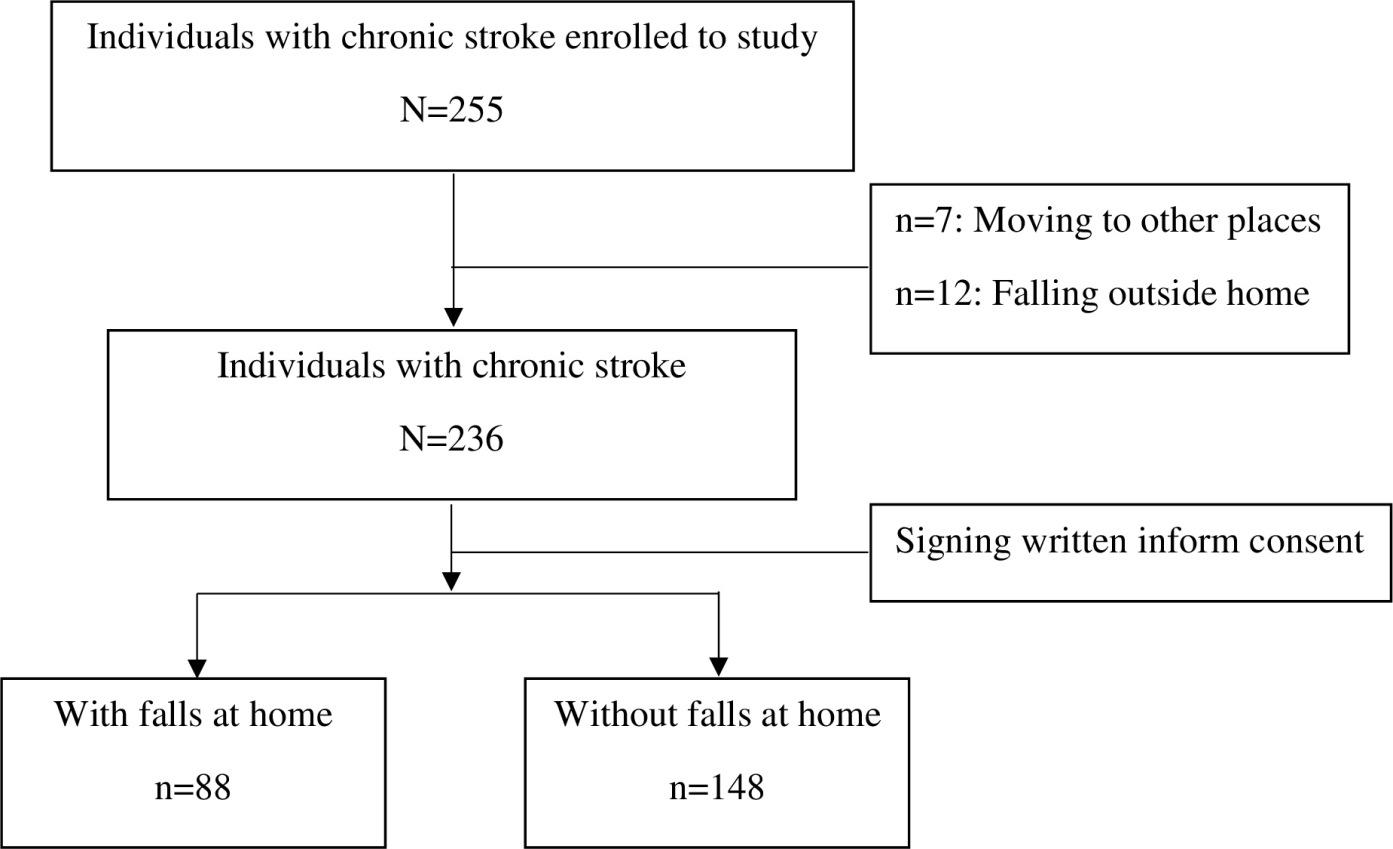
Flow chart of participants.

Two hundred and fifty-five (N = 255) individuals with chronic stroke enrolled on the study. Seven of them moved to other places and 12 fell outside home before participation. Thus, 236 individuals with chronic stroke participated in the study, 88 of them fell at home (37%) and 148 did not fall at home (63%) in the last 6 months prior to participation (Fig 2).

As shown in Table 1, most of participants required some help but able to walk independently with assistive devices (mRS = 3). Participants in the group of falls at home showed significantly greater number of disability (mRS = 4) (p = 0.001), having caregiver (p = 0.012), muscle tone (p<0.0001), TUG (p = 0.001), number of home hazard environments (p<0.0001), and FES (p<0.0001), but demonstrated significantly less FMA-UE (p<0.0001), FMA-LE (p<0.0001), APS (p = 0.003), ST (p<0.0001), BBS (p<0.0001), BI (p = 0.001), SIS-P (p = 0.019), and number of home safety surroundings than those without falls (p<0.0001).

**Table 1:** Characteristics of participants and results of observed variables.

	All	Individuals with chronic stroke		p-value
	(N = 236)	With	Without	
		falls at home	falls at home	
		(n = 88)	(n = 148)	
**Characteristics of participants**				
Age (years)				
Mean ± SD	62.5 ± 10.3	62.4 ± 10.2	61.8 ± 10.4	0.644
Min-Max	30.0–92.0	37.0–91.0	30.0–92.0	
Gender (n, %)				
Male	153 (64.8%)	53 (60.2%)	100 (67.6%)	0.253
Female	83 (35.2%)	35 (39.8%)	48 (32.4%)	
Body mass index (kg/m^2^)				
Median (IQR)	23.9 (4.7)	24.2 (4.5)	24.3 (4.9)	0.964
Min-Max	15.6–38.5	15.6–38.5	15.8–37.7	
Stroke type (n, %)				
Ischemic	171 (72.5%)	66 (75.0%)	105 (70.9%)	0.5
Hemorrhage	65 (27.5%)	22 (25.0%)	43 (29.1%)	
Weakness side (n, %)				
Right	129 (54.7%)	42 (47.7%)	87 (58.8%)	0.099
Left	107 (45.3%)	46 (52.3%)	61 (41.2%)	
Time since stroke (months)				
Median (IQR)	38.5 (81.5)	41.5 (81.8)	36.5 (72.0)	0.386
Min-Max	6.0–264.0	6.0–264.0	6.0–246.0	
Comorbidity (n, %)				
Hypertension	192 (81.4%)	76 (86.4%)	116 (78.4%)	0.128
Diabetes	64 (27.1%)	30 (34.1%)	34 (23.0%)	0.063
Hypercholesteremia	39 (16.5%)	17 (19.3%)	22 (14.9%)	0.373
Others	24 (10.2%)	10 (11.4%)	14 (9.5%)	0.64
Disability degree (n, %)				
mRS = 2	37 (15.7%)	10 (11.4%)	27 (18.2%)	0.001*
mRS = 3	173 (73.3%)	60 (68.2%)	113 (76.4%)	
mRS = 4	26 (11.0%)	18 (20.5%)	8 (5.4%)	
Gait devices (n, %)				
1-point cane	44 (18.7%)	15 (17.0%)	29 (19.5%)	0.091
3 or 4-point cane	97 (41.2%)	42 (47.7%)	55 (37.2%)	
Walker	27 (11.3%)	13 (14.8%)	14 (9.5%)	
None	68 (28.8%)	18 (20.5%)	50 (33.8%)	
Having caregiver (n, %)				
Yes	54 (22.9%)	28 (31.8%)	26 (17.6%)	0.012*
No	182 (77.1%)	60 (68.2%)	122 (82.4%)	
Receiving physical therapy (n, %)				
Yes	57 (24.2%)	24 (27.3%)	33 (22.3%)	0.388
No	179 (75.8%)	64 (72.7%)	115 (77.7%)	
**Observed variables**				
**Structural impairment**				
MAS-EF (scores)				
Median (IQR)	2.0 (2.0)	2.5 (2.8)	1.0 (2.0)	<0.0001*
Min-Max	0.0–5.0	0.0–5.0	0.0–5.0	
MAS-AP (scores)				
Median (IQR)	2.0 (3.0)	3.0 (2.8)	1.0 (2.0)	<0.0001*
Min-Max	0.0–5.0	0.0–5.0	0.0–5.0	
FMA-UE (scores)				
Median (IQR)	37.5 (42.0)	20.0 (42.0)	42.5 (41.0)	<0.0001*
Min-Max	2.0–66.0	4.0–66.0	2.0–66.0	
FMA-LE (scores)				
Median (IQR)	23.0 (11.0)	19.5 (12.0)	24.0 (10.0)	<0.0001*
Min-Max	6.0–34.0	6.0–33.0	6.0–34.0	
FMA-S (scores)				
Median (IQR)	22.0 (5.0)	22.0 (5.0)	22.5 (6.0)	0.87
Min-Max	0–24.0	0–24.0	2.0–24.0	
APS (% of the unaffected APS)				
Median (IQR)	53.0 (55.0)	38.9 (70.9)	55.7 (37.5)	0.003*
Min-Max	0–98.9	0–98.9	0–97.1	
**Activity limitations**				
TUG (seconds)				
Median (IQR)	28.1 (28.8)	36.8 (53.1)	26.1 (20.9)	0.001*
Min-Max	10.7–300.0	13.7–300.0	10.7–130.0	
ST (number of steps)				
Median (IQR)	6.0 (6.0)	5.0 (7.0)	7.0 (4.0)	< 0.0001*
Min-Max	0–15.0	0–12.0	0–15.0	
BBS (scores)				
Median (IQR)	41.0 (16.0)	36.5 (17.0)	45.5 (14.0)	<0.0001*
Min-Max	4.0–55.0	4.0–55.0	4.0–55.0	
BI (scores)				
Median (IQR)	100.0 (5.0)	97.5 (10.0)	100 (5.0)	0.001*
Min-Max	0–100.0	25.0–100.0	0–100.0	
**Participation restrictions**				
SIS-P (scores)				
Median (IQR)	21.9 (39.8)	18.8 (33.6)	25.0 (40.6)	0.019*
Min-Max	0.0–100.0	0.0–81.3	0.0–100.0	
**Contextual factors**				
Home hazard environments (items)				
Median (IQR)	10.0 (8.0)	16.0 (9.0)	9.0 (4.0)	<0.0001*
Min-Max	2.0–33.0	4.0–33.0	2.0–30.0	
Home safety surroundings (items)				
Median (IQR)	12.0 (5.0)	11.0 (4.5)	13.0 (5.0)	<0.0001*
Min-Max	6.0–23.0	6.0–18.0	6.0–23.0	
Risk behaviors (scores)				
Median (IQR)	18.0 (7.0)	17.0 (7.0)	18.0 (8.0)	0.677
Min-Max	11.0–35.0	11.0–35.0	(11.0–31.0)	
FES (scores)				
Median (IQR)	49.5 (53.25)	68.5 (50.8)	38.5 (43.8)	<0.0001*
Min-Max	13.0–130.0	13.0–130.0	13.0–130.0	

*p<0.05 significant difference from individuals with stroke without falls at home.

mRS: Modified Rankin Scale; MAS-EF: modified Ashworth scale on elbow flexor; MAS-AP: modified Ashworth scale on ankle plantarflexor; FMA-UE: Fugl-Meyer Assessment upper extremity function; FMA-LE: Fugl-Meyer Assessment lower extremity function; FMA-S: Fugl-Meyer Assessment sensory function; APS: Ankle plantarflexor strength; TUG: Timed Up and Go Test; ST: Step Test; BBS: Berg Balance Scale; BI: Barthel Index; SIS-P: Stroke Impact Scale-participation/role function; FES: Fall-related Self Efficacy.

### Structural model of falls at home

#### Association between observed variables

Table 2 shows association between observed variables. There was no multicollinearity revealing no collinearity presented by t>0.1 and low correlation among variables presented by small VIF. Only the association between FMA-UE and FMA-LE showed strong correlation coefficient (r_s_ = 0.75), then uniting and naming “FMA-motor (FMA-M)”. Thus, the variables selecting to the measurement model were MAS, FMA-M, FMA-S, APS, TUG, ST, BBS, BI, SIS-P, home hazard environments, home safety surroundings, risk behaviors, and FES.

**Table 2:** Correlation coefficient (r_s_), tolerance (t), and variance inflation factor (VIF) values of a pair of measure variables.

Variables	Statistic parameters	FMA-UE	FMA-LE	FMA-S	APS	TUG	ST	BBS	BI	SIS-P	Home hazard environments	Home safety surroundings	Risk behaviors	FES
**MAS**	r_s_	0.05	0.1	0.003	0.1	0.03	0.11	0.09	0.08	0.08	0.06	0.09	0.15*	0.04
	t	0.44	0.3	0.85	0.46	0.55	0.47	0.91	0.68	0.77	0.88	0.89	0.94	0.48
	VIF	2.3	3.29	1.18	2.16	1.82	2.12	1.09	1.46	1.3	1.14	1.12	1.06	2.07
**FMA-UE**	r_s_		0.75**	0.28**	0.61**	0.42**	0.42**	0.14**	0.23**	0.18**	0.14*	0.07	0.04	0.28**
	t		0.39	0.85	0.45	0.55	0.43	0.91	0.69	0.77	0.88	0.89	0.49	0.93
	VIF		2.56	1.18	2.22	1.82	2.12	1.1	1.45	1.31	1.14	1.21	2.05	1.07
**FMA-LE**	r_s_			0.33**	0.70**	0.51**	0.53**	0.15*	0.25**	0.13*	0.09	0.06	0.05	0.34**
	t			0.85	0.38	0.55	0.49	0.91	0.68	0.77	0.87	0.9	0.93	0.49
	VIF			1.18	2.64	1.81	2.03	1.1	1.47	1.31	1.15	1.11	1.08	1.08
**FMA-S**	r_s_				0.28**	0.26**	0.25*	0.03	0.1	0.15*	0.08	0.05	0.04	0.21**
	t				0.85	0.55	0.48	0.91	0.69	0.76	0.88	0.89	0.92	0.48
	VIF				1.18	1.83	2.09	1.1	1.46	1.31	1.14	1.12	1.08	2.7
**APS**	r_s_					0.36**	0.38**	0.09	0.13	0.07	0.09	0.07	0.003	0.20**
	t					0.55	0.48	0.91	0.68	0.76	0.87	0.9	0.93	0.48
	VIF					1.82	2.1	1.1	1.47	1.32	1.15	1.12	1.08	2.06
**TUG**	r_s_						0.69**	0.21**	0.50**	0.35**	0.04	0.19**	0.06	0.61**
	t						0.52	0.92	0.7	0.76	0.87	0.91	0.5	0.93
	VIF						1.93	1.09	1.44	1.31	1.15	1.11	2.01	1.08
**ST**	r_s_							0.16*	0.36**	0.26**	0.06	0.23**	0.1	0.58**
	t							0.91	0.69	0.76	0.87	0.91	0.93	0.54
	VIF							1.1	1.45	1.32	1.15	1.11	1.08	1.84
**BBS**	r_s_								0.15*	0.11	0.03	0.02	0.08	0.16
	t								0.68	0.76	0.87	0.9	0.93	0.48
	VIF								1.47	1.31	1.14	1.11	1.08	2.07
**BI**	r_s_									0.31**	0.22**	0.24**	0.03	0.53**
	t									0.76	0.92	0.9	0.92	0.54
	VIF									1.31	1.09	1.12	1.08	1.84
**SIS-P**	r_s_										0.02	0.17**	0.04	0.46**
	t										0.87	0.9	0.92	0.53
	VIF										1.51	1.12	1.08	1.89
**Home hazard environments**	r_s_											0.04	0.08	0.01
	t											0.9	0.94	0.48
	VIF											1.11	1.06	2.07
**Home safety surroundings**	r_s_												0.13*	0.15*
	t												0.94	0.48
	VIF												1.07	2.07
**Risk behaviors**	r_s_													0.07
	t													0.92
	VIF													1.08

* p<0.05,

** p<0.01 Significant correlation.

MAS: modified Ashworth scale; FMA-UE: Fugl-Meyer Assessment upper extremity function; FMA-LE: Fugl-Meyer Assessment lower extremity function; FMA-S: Fugl-Meyer Assessment sensory function; APS: Ankle plantarflexor strength; TUG: Timed Up and Go Test; ST: Step Test; BBS: Berg Balance Scale; BI: Barthel Index; SIS-P: Stroke Impact Scale-participation/role function; FES: Fall-related Self Efficacy.

#### Convergence of the first order measurement model

**Fig 3:**
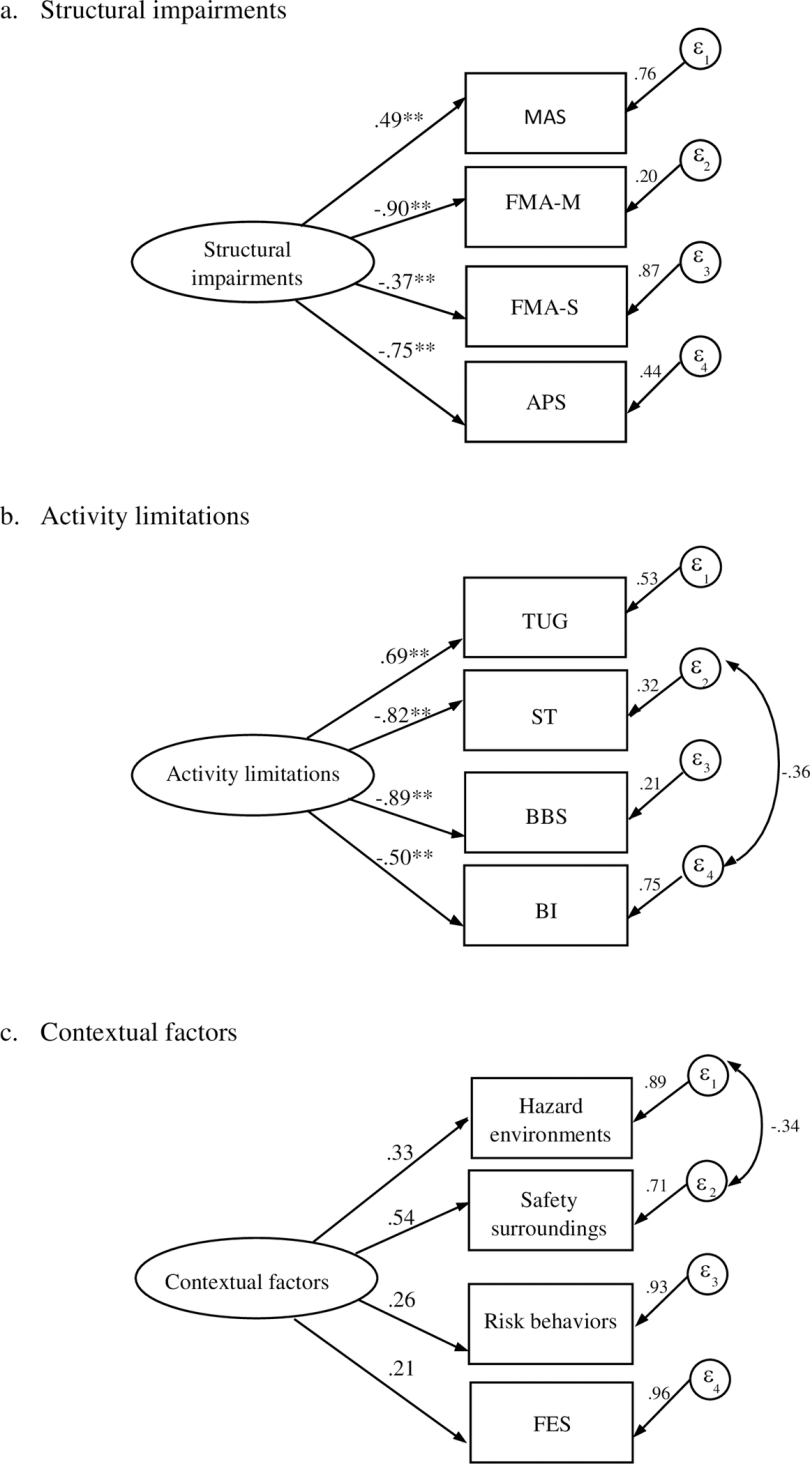
Confirmatory factor analysis of the first order measurement model: a. structural impairments, b. activity limitations, and c. contextual factors. Value of factor loading and amount of measurement error are shown. **p < 0.001. MAS: modified Ashworth scale; FMA-M: Fugl-Meyer Assessment upper and lower extremity function; FMA-S: Fugl-Meyer Assessment sensory function; APS: Ankle plantarflexor strength; TUG: Timed Up and Go Test; ST: Step Test; BBS: Berg Balance Scale; BI: Barthel Index; SIS-P: Stroke Impact Scale-participation/role function; FES: Fall-related Self Efficacy; εn: residual variable.

The MAS, FMA-M, FMA-S and APS were accurate measurements for the structural impairments domain (χ2 = 0.32, df = 2, p = 0.85, χ2/df = 0.16, CFI = 1.00, RMSEA = 0.00, SRMR = 0.01). Among these variables, FMA-M and APS exhibited a high relationship to the domain showing -0.90 and -0.75, respectively. The MAS showed a positive relationship to structural impairments, whereas the FMA-M, FMA-S and APS demonstrated the negative association (Fig 3A).

The TUG, ST, BBS, and BI did not fit to the initial model of activity limitations (χ2 = 9.28, df = 2, p = 0.10, χ2/df = 4.64, CFI = 0.94, RMSEA = 0.12, SRMR = 2.04), then revising with modification index value of 9.05 that was covariance between ST and BI and finding a good fit to the model (χ2 = 0.24, df = 1, p = 0.63, χ2/df = 0.24, CFI = 1.00, RMSEA = 0.00, SRMR = 0.01). The TUG, ST, BBS, and BI were high correlation to the model, with the ST and BBS being the maximum two. The TUG was positively related to activity limitations, whereas the ST, BBS and BI were negative relation (Fig 3B).

Home hazard environments, home safety surroundings, risk behaviors, and FES did not fit to the initial model (χ2 = 6.28, df = 2, p = 0.04, χ2/df = 3.14, CFI = 0.56, RMSEA = 0.10, SRMR = 0.05), then revising with modified index value of 5.42 that was covariance between home hazard environments and safety surroundings and finding a good fit to the contextual factors model (χ2 = 0.58, df = 1, p = 0.36, χ2/df = 0.58, CFI = 1.00, RMSEA = 0.00, SRMR = 0.01). However, those variables showed no significant association with the model (Fig 3C).

#### Convergence of the second order measurement model

**Fig 4:**
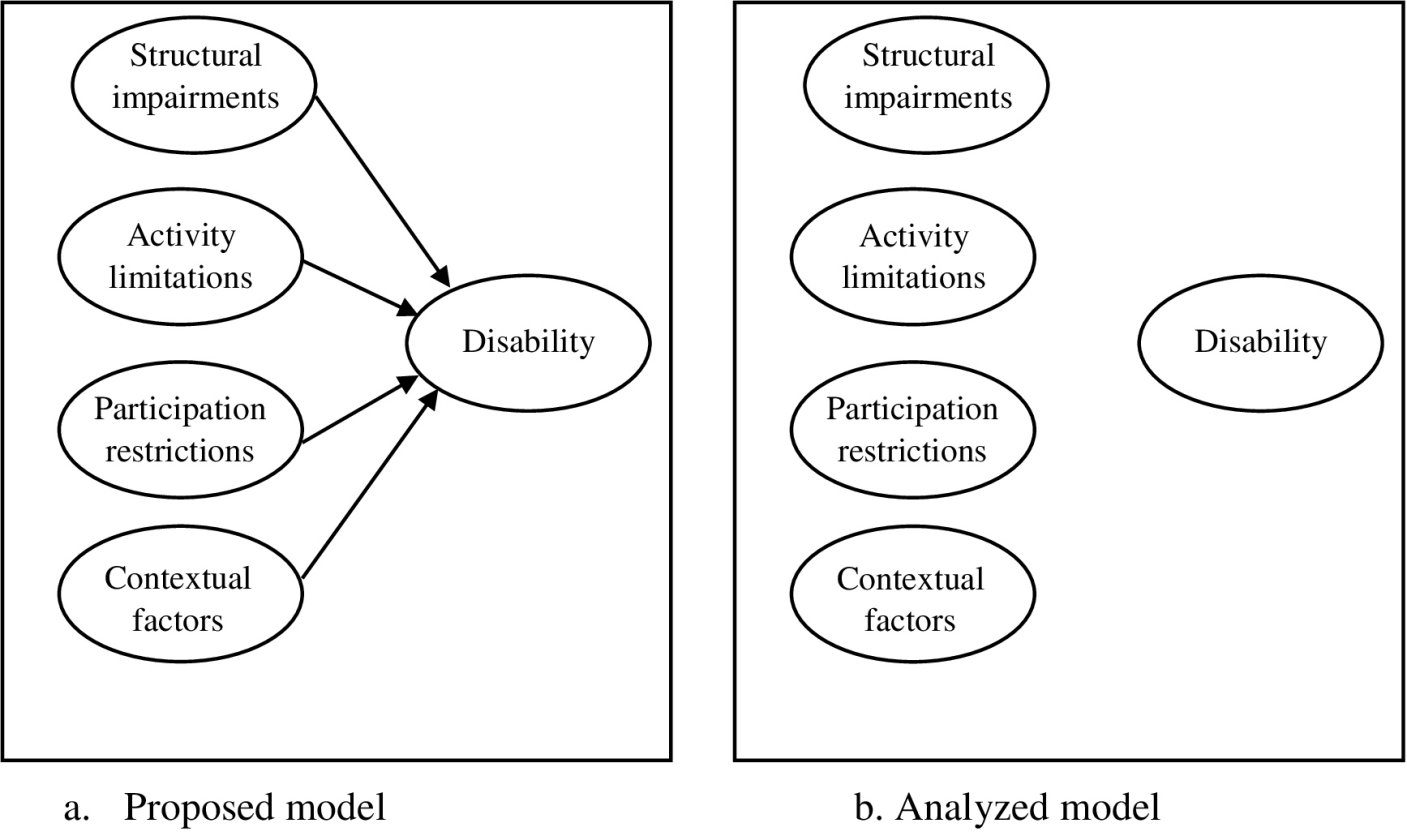
Confirmatory factor analysis of the second order measurement model. In the hypothesis model it was proposed for convergence of structural impairments, activity limitations, participation restrictions, and contextual factors toward disability (a). The analysis showed failed model (b).

The structural impairment, activity limitations, participation restrictions, and contextual factors toward disability did not achieve the criteria of goodness of fit indexes (χ2 = 764.08, df = 74, p = 0.00, χ2/df = 10.33, CFI = 0.02, RMSEA = 0.90, SRMR = 1.99). Although the model modification technique was used by modification index value of 1.07 that was covariance between structural impairments and activity limitations, the CFA did still not demonstrate the convergence feature (χ2 = 763.01, df = 74, p = 0.00, χ2/df = 10.27, CFI = 0.02, RMSEA = 0.90, SRMR = 1.99), as shown in Fig 4.

#### Structural model

**Fig 5:**
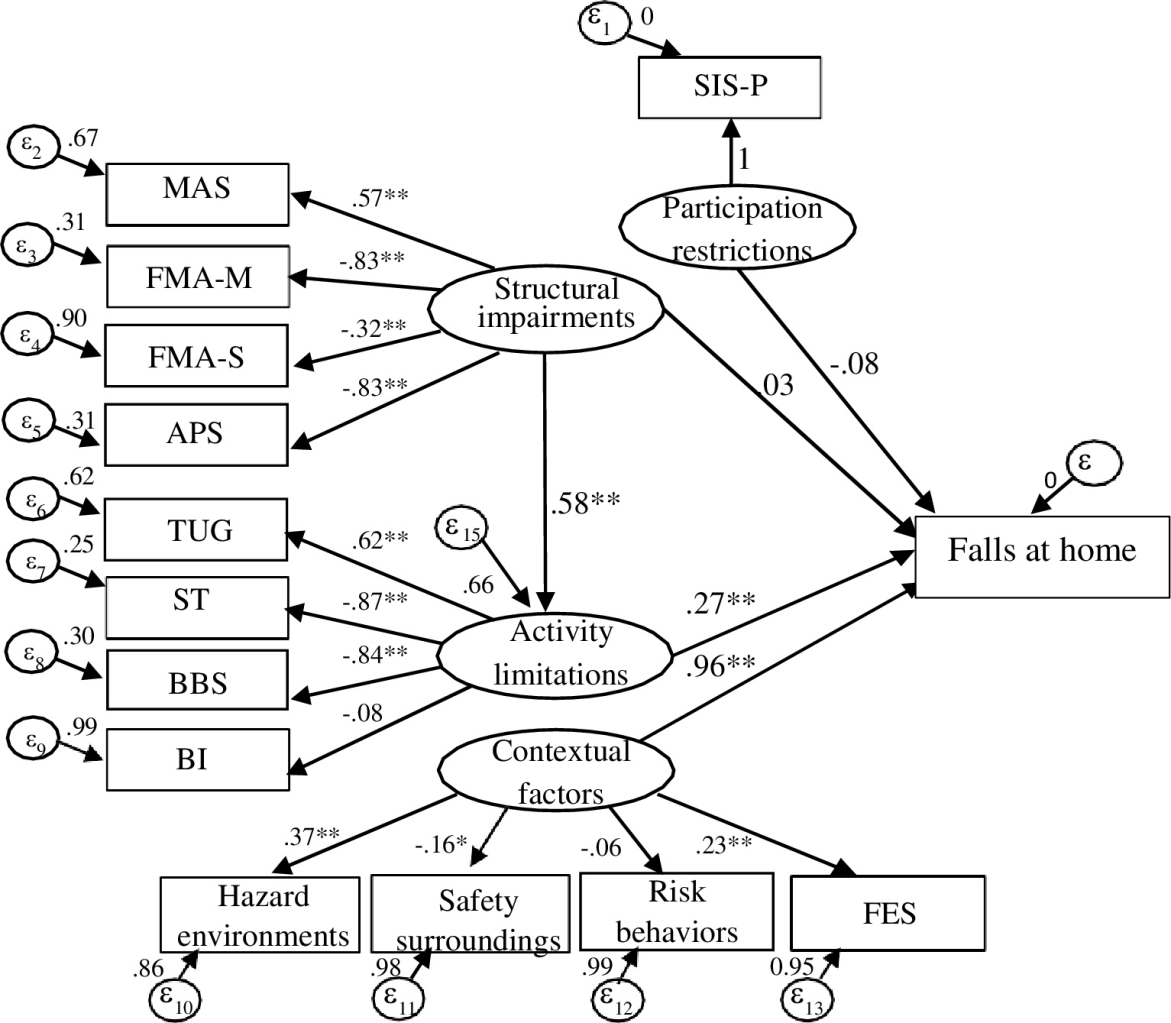
The structural model of falls at home in individuals with stroke. Value of factor loading and amount of measurement error are shown. The inter-relationships of residual variables are not shown. *p < 0.05, **p < 0.001. MAS: modified Ashworth scale; FMA-M: Fugl-Meyer Assessment upper and lower extremity function; FMA-S: Fugl-Meyer Assessment sensory function; APS: Ankle plantarflexor strength; TUG: Timed Up and Go Test; ST: Step Test; BBS: Berg Balance Scale; BI: Barthel Index; SIS-P: Stroke Impact Scale-participation/role function; FES: Fall-related Self Efficacy; εn: residual variable.

Since the second order measurement model was poor fit, the hypothesis model was revised. The structural model suggested direct relationship of the structural impairments, activity limitations, participation restrictions, and contextual factors with falls at home. Additionally, indirect effect of structural impairments with falls at home was shown through activity limitations, whereas other interrelationships between domains assuming in the hypothesis model were removed. The new model of falls at home in individuals with chronic stroke is proposed (Fig 5).

Fig 5 describes the structural model of falls at home in individuals with chronic stroke. The first SEM analysis indicated that the model did not fit with the sample data (χ2 = 333.95, df = 76, p = 0.00, χ2/df = 4.39, CFI = 0.62, RMSEA = 0.12, SRMR = 1.39). Therefore, the model was adjusted and demonstrated the acceptable model (χ2 = 96.58, df = 51, p = 0.00, χ2/df = 1.89, CFI = 0.93, RMSEA = 0.06, SRMR = 0.04).

The activity limitations and contextual factors demonstrated significant (p<0.01) paths with falls at home. The standardized path coefficients for activity limitations and contextual factors were 0.27 (95%CI: 0.17, 0.37) and 0.96 (95%CI: 0.93, 0.99), respectively. In contrast, the structural impairments and participation restrictions did not show a path with falls at home. The standardized path coefficients for structural impairments and participation restrictions were 0.03 (95%CI:-0.05, 0.12) and -0.08 (95%CI: -0.07, 0.05), respectively. However, structural impairments demonstrated a significant (p<0.01) path with activity limitations (coefficient: 0.58; 95%CI: 0.47, 0.69) and some distribution to falls at home through activity limitations (0.58x0.27 = 0.16).

The combination of contextual factors with structural impairments, activity limitations and participation restrictions in the structural model showed that the contextual factors were the strongest path on falls at home (coefficient = 0.96). A good example of relationship characteristics was observed in the covariance between the measures in contextual factors and those in structural impairments and activity limitations. The MAS, FMA-M, FMA-S, APS measuring structural impairments, the TUG, ST, BBS measuring activity limitations, the home hazard environments, home safety surroundings, and FES measuring contextual factors were related to falls at home.

## Discussion

The present study explained a model of falls at home in individuals with chronic stroke. The structural impairments influenced falls at home through activity limitations. The activity limitations and contextual factors directly related to falls at home, that the contextual factors were the strongest influence showing an impact of home hazard environments, home safety surroundings, and fall concern combining to MAS, FMA-M, FMA-S, APS, TUG, ST, BBS.

Participants were individuals with chronic stroke and moderate disability. About 37% reported falls at home in the last 6-month period. The rate was close to 33% and 40% reported for individuals with chronic stroke with a fall during 1-year time [10], and within a 6-month period [5], respectively. However, the rate in the present study was lower than 46% reported for individuals with stroke falling in the first 6 months after discharge from rehabilitation centers to home [56] and smaller than 76% reported for individual with chronic stroke falling in the period of 80 months since stroke [57]. The lower incidence in the present study could be a result of the shorter period of observation. However, the rate in the present study was higher than 25% reported for individuals with stroke who fell observed over 6 months [58]. The present study observed falls at home where most participants did activities by themselves, as shown approximately 70% of fallers demonstrating no caregivers, and about 80% could transfer and ambulate independently with gait aids, whereas the previous study [58] investigated falls both inside and outside home and showed that almost half of their participants used wheelchair for ambulation, and their participants might receive a close nursing of caregivers that might decrease chance of falling. Therefore, the percentage of participants with falls at home the present study was reasonable.

### Association between observed variables

A strong relationship between FMA-UE and FMA-LE was found. The association has been reported and revealed the existence of a connection between hierarchical recovery components of upper and lower extremity in individuals with chronic stroke [59]. A study exploring association between arm function and postural balance suggested to concern not only lower limb but also the upper influencing on postural balance [13]. With the significant relationship establishing in the method and the evidence-based information it is definite to combine FMA-UE and FMA-LE to become a single variable for the next step of analysis. The present study also observed association between other pairs among variables of structural impairments, activity limitations, participation restrictions, and contextual factors; for example, MAS and risk behavior, FMA-UE and SIS-P, FMA-UE and TUG, FMA-LE and BBS, APS and FES, SIS-P and BI. The association between FMA- either UE or LE and BBS as well as TUG was reported in individuals with stroke [13]. However, in the present study the correlation was out of the established importance boundary. Thus, those pairs could not develop to be one variable. The present finding found no correlation in most of the variables between structural impairments and contextual factors such as MAS and home hazard environments, FMA- either UE, LE, or ST and risk behaviors. The result was consistent to a meta-analysis showing no association between motor impairment and fall risk in individuals with stroke, discussing high heterogeneity of measure use [60]. Although there were several correlation pairs in the present study, only one pairs showed strong relationship and could merged to one variable according to the statistical setting criteria.

### Measurement model

The first order measurement model demonstrated involvement of the MAS, FMA-M, FMA-S and APS in the structural impairments model, and association of the TUG, ST, BBS, and BI in the activity limitations model. In contrast, home hazard environments, home safety surroundings, risk behaviors, and FES showed disassociation to the contextual factors model. A previous review reported a good agreement in MAS and FMA for measuring body function domain in the ICF for individuals with stroke [21]. The present finding corresponds to the knowledge. Additionally, the present result indicates that motor function domain of the FMA is a good measure for structural impairments, but sensory function may be less suitably. Although the APS was not generally mentioned as a body function measure or stated in a previous review [21], the present finding demonstrated high relationship of the APS in the model. An explanation may be the important of the APS in walking speed [27, 28], walking line [29], and foot pressure [29] among individuals with stroke. It is, therefore, suggested to consider the APS as an essential measure for structural impairments in individuals with stroke. In the present structural impairments model, the MAS, FMA-M, FMA-S and APS are acceptable to be variables.

The initial model of activity limitations was incomplete, however, the model was satisfied after mobilization with covariance between ST and BI. The ST and BI may not be a reasonable measure. Furthermore, in the existing situation there may be unspecified factors. The ST and BI are quite difference measurement. The ST assesses standing balance on the affected leg [38], but the BI examines activities of daily living using both the affected and unaffected upper and lower limbs [43]. However, in the activity limitations model, the ST, BI, TUG, and BBS were important connection. The BBS and BI was recommended for measuring activity in the ICF [22]. The present finding agrees with the previous report [22] and revealed that the BBS showed the highest correlation, while the BI demonstrated the lowest. Although the BBS and BI evaluate performance in everyday life, they are difference in features. The BBS assesses maintaining and changing postural tasks [48], whereas the BI evaluates independence of care for oneself [43]. Similar to the BBS, the TUG [47] and ST [38] examine movement task requiring balance. Both measures appeared to produce higher correlation in the model than the BI. The explanation may be the related task among the TUG, ST, and BBS. No matter what the TUG, ST, BBS, and BI evaluate, they are suitable for the activity limitations model.

The present study showed no relationship of home hazard environments, home safety surroundings, risk behaviors, and FES to the contextual factors. There are several measurements for environmental factors such as Craig Hospital Inventory of Environment Factors [61], Facilitator and Barriers Survey/Mobility [62], however, the instruments remain intangible [63]. A new measure, the Environmental Factors Item Banks [64], has been developed and examines environmental chapters of the ICF. Nonetheless, the measure was inappropriate to the present study investigating only home environments. The current study used survey of home hazard environment and safety surroundings to direct the effect of environment on participation of participants. Similarly, risk behavior and FES were completely reported by participants. To our knowledge, there was no common falls measure for personal factors of the ICF. In the present study the FES was chosen since it is a well-known self-measurement concerning falls [44]. However, home hazard environment, home safety surroundings, risk behavior, and FES appeared not to represent or could not directly measure the contextual factors in the first order measurement model in the present study.

The second order measurement model revealed the fail model. According to the ICF, disability is affected by structural impairments, activity limitations, participation restrictions, and contextual factors individually or corporately [17]. No direct association was found in the model that may result from the relationship between the domains, or the domains representing different issues. Therefore, structural impairments, activity limitations, participation restrictions, and contextual factors were declined to the disability in the second order measurement model. As a result, the domains were directly connected to falls at home in the structural model.

### Structural model

The initial structural model of falls at home was a poor fit that may be from interrelated factors or inappropriate measures in the model. However, after mobilization, the final model demonstrated the acceptable model. The contextual factors appeared to be a strong direct association with falls at home. The activity limitations were also straight to the falls, but the structural impairments affected falls at home via the activity limitations. The association between structural impairments and activity limitations has been reported in individuals with chronic stroke, for example, combination of trunk and leg muscle strength relating to standing [65], and also proposed strength training in the trunk and leg for balance intervention to reduce fall risk and improve activities of daily living [65].

Although home hazard environments, home safety surroundings, risk behaviors, and FES showed no relationship with the contextual factors in the first measurement model, these variables, except risk behaviors, appeared to be measures of the contextual factors in the structural model. It is, therefore, that home hazard environments, home safety surroundings, and FES are required to consider among other measures of structural impairments, activity limitations, and participation restrictions. Home hazard environments induced falls at home in individuals with chronic stroke. Indoor tripping exposures are a risk factor for falls in community-dwelling individuals with stroke [11]. In the present study, indoor step, slippery surface or mat, and scatter rugs were found as dangerous environments in home. Another essential factor relating to falls at home was home safety surroundings. Participants were found to use a wall, table, cupboard, and pole, instead of gait aids, to support transferring and ambulation in home, similarly to a study reporting catching the nearby objects to recover balance [8]. No or infrequent use of walking devices may be due to insufficient space or messy room. Several home safety techniques are recommended for individuals with stroke, for instance, rearranging furniture, fixing grab bars, railing stairs, and removing scatter rugs [66]. Home adjustments for individuals with stroke have been made and maintain to the demand that is used for years after stroke to avoid falling and provide safety for essential mobility [67]. It may increase confidence in ability to avoid falling during engaging in everyday activities. Therefore, FES was possible to involve in the structural model of falls at home. Individuals with stroke have impairments and activity limitations, for example, increased muscle tone disturbing functional movements [68, 69], resulting in high risk for falling [3, 4, 70, 71]. To prevent falls at home, home hazard environments are removed and home safety surroundings are provided, as found positively and negatively in the structural model, respectively. Concurrent organization of structural impairments, activity limitations, and contextual factors for individuals with stroke is needed to avoid falling in doing activities.

The BI, a measure of activity limitations, was not associated with its domain in the structural model of falls at home. An assistance of caregivers in doing activities of daily living might be an explanation. In contrast, the TUG, ST, and BBS was related to activity limitations in the structural model. It may be participants performing the tests by themselves and the measures assessing balance. The impaired balance is a common cause of falling after stroke [57, 72] and demonstrated low quality of life [57]. It is, therefore, no doubt to observe association of TUG, ST, and BBS in the structural model. The present study suggests that postural balance components are important to falls at home in the structural model.

Besides describing in the first order measurement model, the MAS, FMA-M, FMA-S, and APS explained falls at home in the structural model, but illustrating through activity limitations. In individuals with stroke, structural impairments such as increased muscle tone [6, 7], impaired upper and lower limbs functions [13, 73–75], decreased sensory functions [74], and reduced APS [76] are risk for falling. The indirect effect of structural impairments may rationalize that in individuals with stroke muscle tone, motor and sensation functions, and APS alone could not affect falls at home. The impairments would be relevant to falls if individuals with stroke did activities. Additionally, the low relationship of FMA-S in the model might reduce power of the structural impairments directly associating with falls at home in the structural model. Therefore, the MAS, FMA-M, FMA-S, and APS influenced on falls at home by means of performing activities.

The present study measure participation restrictions by one observed variable, the SIS-P, and it did not influence on falls at home in the structural model. The SIS-P is a reliable measurement assessing participation perception both inside and outside home activities in individuals with chronic stroke and mild to moderate disability [77]. The present study recruited only individuals with chronic stroke falling inside home. Hence, the SIS-P may not fulfill with falls at home in the structural model. It is difficult to obtain a suitable measure for participation restrictions because measures for participation restrictions need agreement in measurement domains, which much emphasize on health-related quality of life, and combine dimensions from structural impairments, activity limitations, and participation restrictions [23]. However, the present finding implies that the SIS-P may be an inappropriate measure and did not influence to falls at home in the model.

The structural model of falls at home indicated that contextual factors had the greatest direct influence on falls at home for individuals with chronic stroke. Home safety assessment and delivery of occupational therapist to inspect home by using an appropriate instrument are recommended [66]. The present finding agrees and suggests to adjust or modify home suitably for lifestyles of individuals with stroke before they are discharged home. Furthermore, home assessment should be done regularly since structural impairments of individuals with chronic stroke may be improved [78] or declined that could influence ability in doing activities. Therefore, appropriate home environments support self-confidence of individuals with chronic stroke in doing tasks at home. This may have important consequences regarding independent living, fall prevention, decreased fear of falls, and damage from falling.

Although, in the structural model, falls at home were recorded before assessment of observed variables, the model could explain falls at home in real situations and may envisage the falls. Since participants were chronic stroke and 70% of them did not receive physical therapy, their structural impairments and activity limitations may not be changed in 6-month period of falls recording. The opinion was supported by a previous study demonstrating recovery of movement and balance ability in individuals with chronic stroke receiving home-based physical therapy periodically [78]. Hence, the structural model of falls at home in the present study could describe cause of falls at home in individuals with chronic stroke.

### Study limitations

There are limitations to the present study. Firstly, sample size was small. The final models had to be adjusted prior to be the final complete model. A larger sample size may be required to lead to better fit indices. Secondly, investigation was conducted in individuals with chronic stroke living and falling inside home. The structural model of falls at home may not be able to apply to those who were acute or subacute stroke, did not live at home, or lived at home but fell outside home. In addition, the study excluded individuals with chronic stroke who had less than 24 points of MMSE and some higher brain dysfunction such as unilateral neglect, unable to communicate, visual impairments. Thus, the model may not explain falls at home in those who have cognitive impairment or higher brain dysfunctions. Thirdly, observed variables of contextual factors were examined in Thai manners. The model of falls at home may have limited generalizability. To increase the generalizability of the model, the future study should investigate in the multi-countries. Fourthly, there was one observed variable of participation restrictions that could not distinguish diversity of participations. Other participation restrictions measures, such as Medical Outcome Study Short Form 36, Stroke Specific Quality of Life [23] are suggested to apply in the model. Lastly, observed variables in the structural model of falls at home were physical factors. Not only physical issues but also mental aspects, cognitive impairments and depression, for example, affect falls in individuals with stroke [60]. It is suggested to include psychological components in future research.

## Conclusions

The structural model of falls at home in individuals with chronic stroke revealed the direct influence of contextual factors and activity limitations but the indirect effect of structural impairments on fall at home. The contextual factors appeared to be the strongest association with falls at home. The structural impairments involved falls at home through activities. Home hazard environments, home safety surroundings, confidence in doing tasks, postural balance, muscle tone, motor and sensory function, and ankle plantarflexor strength were weighed together to influence on falls at home. It is suggested to arrange home appropriately to the structural impairments and activity limitations of individuals with chronic stroke to allow them to optimum functioning without risk of falls.

## Supporting information

S1 Data (PDF)

S2 Data (PDF)

S3 Data (PDF)

S4 Data (PDF)
