# Supplementary material for: A structural equation model of falls at home in individuals with chronic stroke, based on the international classification of function, disability, and health
Source: PLoS One. 2020 Apr 10;15(4):e0231491. doi: 10.1371/journal.pone.0231491 (PMC7147784; doi:10.1371/journal.pone.0231491)
Supplement: S2 Data — (PDF) [file pone.0231491.s002.pdf]

Risk behavior assessment questionnaire

In the last 6 months, how often have you performed these activities after stroke?

Activities	Frequency of doing activity			
	Never	Sometimes	Often	Always
1) Hurry to stand up or sit.	☐	☐	☐	☐
2) Sitting down on the floor or standing up from the floor.	☐	☐	☐	☐
3) Picking item which is above the eye level from the shelf.	☐	☐	☐	☐
4) Picking objects from the floor.	☐	☐	☐	☐
5) Standing to do activities requiring 2 hands, such as washing dishes, cooking foods.	☐	☐	☐	☐
6) Standing for dressing, such as wearing pants or shirt, or combing while standing.	☐	☐	☐	☐
7) Doing activities that you have never done by yourself and did not wait for caregiver/helper.	☐	☐	☐	☐
8) Walking with fast speed.	☐	☐	☐	☐
9) Walking without a walking aid even though you usually use it.	☐	☐	☐	☐
10) Walking with carrying object in 2 hands.	☐	☐	☐	☐
11) Going to the bathroom at night without turning on the light.	☐	☐	☐	☐

Kalaya Kongwattanakul & Vimonwan Hiengkaew, Faculty of Physical Therapy, Mahidol University, 2020.
