# Supplementary material for: A structural equation model of falls at home in individuals with chronic stroke, based on the international classification of function, disability, and health
Source: PLoS One. 2020 Apr 10;15(4):e0231491. doi: 10.1371/journal.pone.0231491 (PMC7147784; doi:10.1371/journal.pone.0231491)
Supplement: S4 Data — (PDF) [file pone.0231491.s004.pdf]

แบบสอบถามพฤติกรรมเสี่ยงต่อการล้ม

ในช่วง 6 เดือน ที่ผ่านมาท่านทำเคยทำกิจกรรมเหล่านี้หลังจากเป็นโรคหลอดเลือดสมองหรือไม่

**กิจกรรม**	**ความถี่ของการทำกิจกรรม**			
	**ไม่เคย**	**นาน ๆ ครั้ง**	**เกือบทุกครั้ง**	**เป็นประจำ**
1) รีบลุกขึ้นยืน หรือนั่ง	☐	☐	☐	☐
2) นั่งลงบนพื้นหรือลุกขึ้นยืนจากพื้น	☐	☐	☐	☐
3) หยิบของจากชั้นที่สูงกว่าระดับสายตา	☐	☐	☐	☐
4) ก้มตัวเพื่อหยิบของจากพื้น	☐	☐	☐	☐
5) ยืนทำกิจกรรมที่ต้องใช้ 2 มือ เช่น ล้างจาน ทำกับข้าว	☐	☐	☐	☐
6) ยืนแต่งตัว เช่น ยืนใส่กางเกง ยืนใส่เสื้อ	☐	☐	☐	☐
7) ทำกิจกรรมที่ไม่เคยทำด้วยตนเองโดยลำพัง ไม่รอผู้ช่วยเหลือ	☐	☐	☐	☐
8) เดินเร็วๆ	☐	☐	☐	☐
9) เดินโดยไม่ใช้อุปกรณ์ช่วยเดิน (โดยปกติจะใช้เป็นประจำ)	☐	☐	☐	☐
10) เดินโดยถือของทั้ง 2 มือ	☐	☐	☐	☐
11) เข้าห้องน้ำในเวลากลางคืนโดยไม่เปิดไฟทางเดิน	☐	☐	☐	☐

กัลยา ก้องวัฒนกุล และ วิมลวรรณ เหียงแก้ว คณะกายภาพบำบัด มหาวิทยาลัยมหิดล 2563
